# Quantitative Relationship Between Color Parameters and Mechanical Property of Epoxy Resin During Thermo-Oxidative Aging

**DOI:** 10.3390/polym18101182

**Published:** 2026-05-12

**Authors:** Geng Hou, Zhenzhong Sun

**Affiliations:** 1 School of Mechanical Engineering, Dongguan University of Technology, Dongguan 523808, China; hougeng@dgut.edu.cn; 2 School of Materials Science and Engineering, Xi’an Jiaotong University, Xi’an 710049, China

**Keywords:** color, strength, epoxy resin, thermo-oxidative aging

## Abstract

This study aims to establish a quantitative relationship between the color parameters and mechanical properties of thermo-oxidatively aged epoxy resin, with the goal of exploring a low-cost, rapid method for mechanical performance assessment based on color measurement. Epoxy resin specimens were subjected to high-temperature aging for varying durations, after which multiple color parameters were measured using a portable colorimeter. The variations in these parameters with aging duration and intensity were systematically characterized. The results indicate that during thermo-oxidative aging, strength exhibits a monotonic correlation with certain color parameters, such as lightness and hue angle. Based on this finding, an empirical model was developed to estimate strength from color parameter values. A comparison between estimated and experimental results confirms the feasibility and potential of this approach. To make the validation more convincing, it utilized not only the data from this experiment but also data from the literature. This work provides a theoretical basis and a practical technical pathway for utilizing portable colorimeter to rapidly and non-destructively assess the aging extent and mechanical performance of polymeric engineering structures.

## 1. Introduction

Epoxy resin is a representative polymeric material. Polymers serve as both essential traditional engineering materials and a core component of modern structural materials. Owing to their favorable toughness, wear resistance, lightweight, and corrosion resistance [1,2,3], they play an indispensable role across a wide range of fields, including mechanical engineering [4,5,6], transportation [7,8,9], aerospace [10,11], civil engineering [12,13,14], medical devices [15,16], and electronic information [17,18]. However, during long-term service, polymers are susceptible to environmental factors, leading to aging that induces changes in molecular and morphological structures [19]. Consequently, their mechanical properties degrade, often manifesting as reductions in strength and toughness [20,21]. For load-bearing polymer components, prolonged aging can directly compromise structural safety and service life. Similar degradation in mechanical performance is also observed in fiber-reinforced polymer composites [22,23,24]. Therefore, exploring characterization and evaluation methods for polymer aging behavior is of significant importance for assessing polymer durability and service life, as well as developing new high-performance polymers.

Studies indicate that environmental factors such as heat [25], electromagnetic radiation [26], light [27], oxygen [28], or moisture [29] can contribute to polymer aging. During the aging process, oxidation initially occurs at free radical sites on molecular chains, subsequently triggering reactions such as chain scission and cross-linking [30]. This process is accompanied by the formation of chromophores, leading macroscopically to a darkening in polymer color [31]. This phenomenon of color deepening has been observed in the thermo-oxidative aging of various polymers, such as nylon [32,33], polypropylene [34,35], and the epoxy resin used in this study [36,37,38]. While these studies have identified and elucidated the mechanisms behind the color change in aged polymers, their analyses remain largely qualitative and have not established a quantitative relationship between color parameters and mechanical properties. Consequently, it poses challenges for the quantitative assessment and evaluation of degradation in engineering structures.

Given that thermo-oxidative aging can simultaneously induce color changes and mechanical degradation in polymers, it is reasonable to infer that estimating post-aging mechanical properties by establishing a relationship between color parameters and mechanical performance should be feasible. Therefore, this study innovatively analyzes the correspondence between parameters such as lightness and chroma of epoxy resin and its mechanical strength and establishes a quantitative mathematical expression to describe this relationship. For practical polymer-based engineering structures, a handheld portable colorimeter [39,40] can be used to measure the lightness and chroma of aged components. Based on the quantified relationship between color parameters and mechanical properties, strength can then be estimated. This approach offers advantages including the ability to perform non-destructive measurements on large-scale structures and low testing costs. Thus, this research provides theoretical guidance for the low-cost and rapid assessment of the mechanical properties of aged polymers based on color measurement.

## 2. Materials and Testings

### 2.1. Material

**Figure 1:**
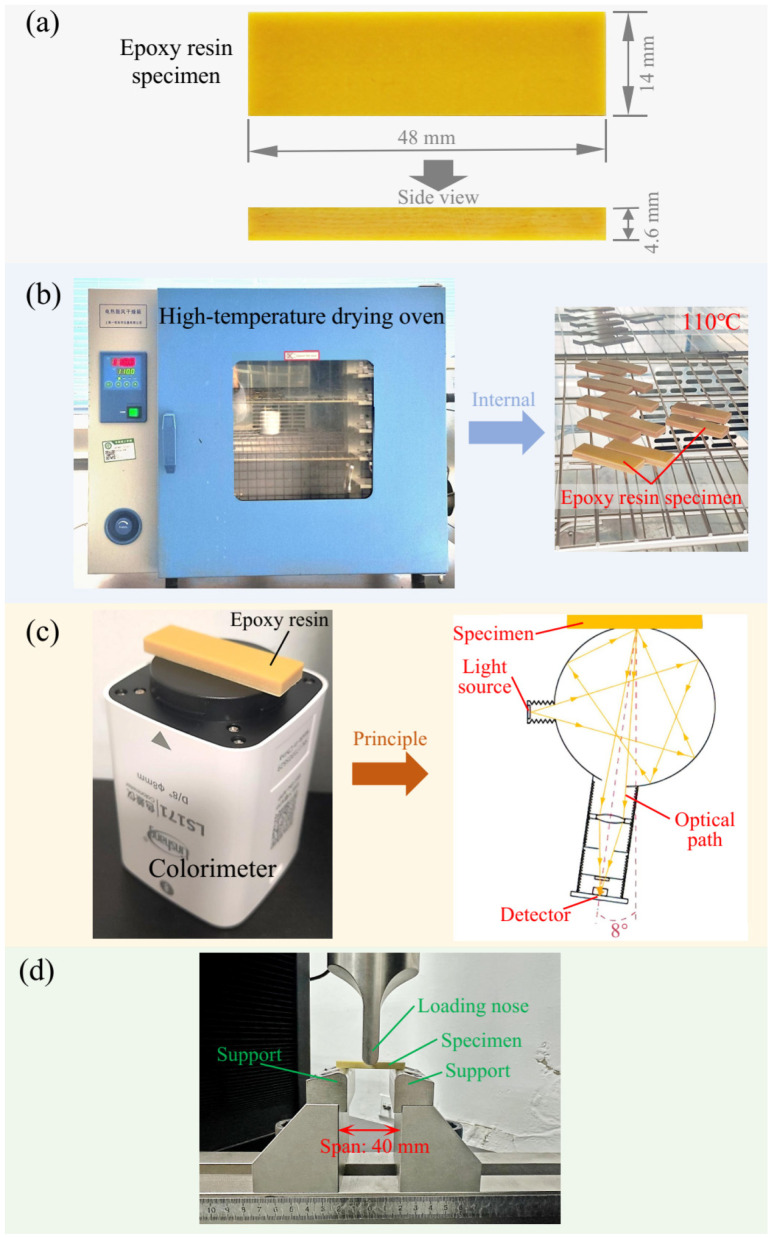
Material and experimental setups used in this study. (**a**) Epoxy resin specimen; (**b**) thermo-oxidative aging test setup; (**c**) method and principle of color parameter measurement [41]; (**d**) three-point bending mechanical property test setup.

The specimens used in this study were made of epoxy resin, with their dimensions and geometry illustrated in Figure 1a. The specimens were plate-shaped cuboids measuring 48 mm × 14 mm × 4.6 mm.

### 2.2. High-Temperature Aging Treatment

To investigate the effects of thermo-oxidative aging on the color and mechanical parameters of epoxy resin, the as-received epoxy resin specimens were subjected to high-temperature aging for varying durations. As shown in Figure 1b, the epoxy resin specimens were placed in an electric drying oven with temperature of 110 °C, and the temperature variation across the specimen placement area is within ±1 °C (see Appendix A). The aging durations include 0, 5, 10, 15, 20, 25, 30, and 35 days. Since noticeable color deepening had already occurred after 35 days of aging, and considering the time cost of the experiment, the maximum aging duration was set to 35 days. Moreover, this duration is sufficient to meet the theoretical research objectives of this work.

### 2.3. Color Parameters Measurement

To quantify the effect of thermo-oxidative aging on the color of the epoxy resin, a portable colorimeter (LS171, Linshang, Shenzhen, China) was used to measure the lightness, chroma, and hue angle of each specimen after high-temperature treatment. The appearance and measurement principle of the colorimeter used are shown in Figure 1c [41]. The instrument employs an 8° optical structure [42] with a D65 light source [42,43] and a 10° field of view. The measurement duration for each specimen was 1 s, with a circular measurement area of 8 mm in diameter. The color parameters were measured at the central region of the specimen, which corresponds to the fracture location in the subsequent three-point bending mechanical performance tests.

### 2.4. Mechanical Property Testing

As shown in Figure 1d, to investigate the effect of thermo-oxidative aging on the mechanical properties of the epoxy resin, three-point bending tests were conducted on the aged specimens using a universal mechanical testing machine in accordance with the ASTM D790-10 standard [44]. From these tests, the bending strength of each specimen was obtained. During testing, the support span was set to 40 mm, and the loading rate of the loading nose was maintained at 0.2 mm/min.

## 3. Results and Discussions

### 3.1. Evolution of Color Parameters with Thermal-Oxidative Aging Duration

**Figure 2:**
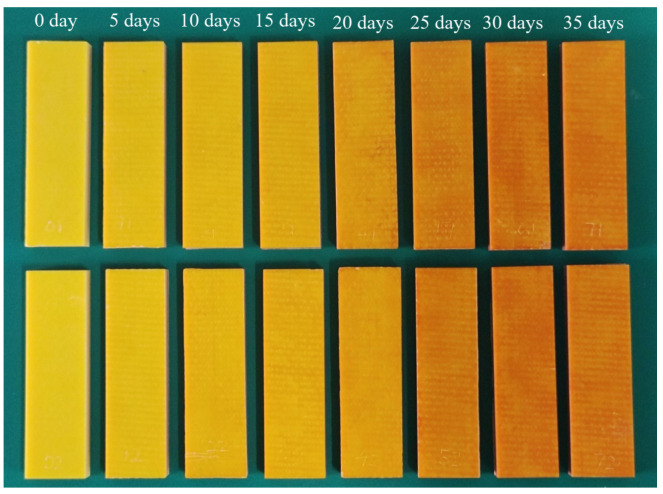
Photos of specimens after different aging durations.

Figure 2 shows photographs of the epoxy resin specimens subjected to thermo-oxidative aging for different durations. It can be observed that the color of the specimens darkens progressively with increased aging duration. However, such qualitative visual inspection is insufficient for accurately determining the degree of color change. Therefore, color quantification using a colorimeter is essential and provides an effective approach.

**Figure 3:**
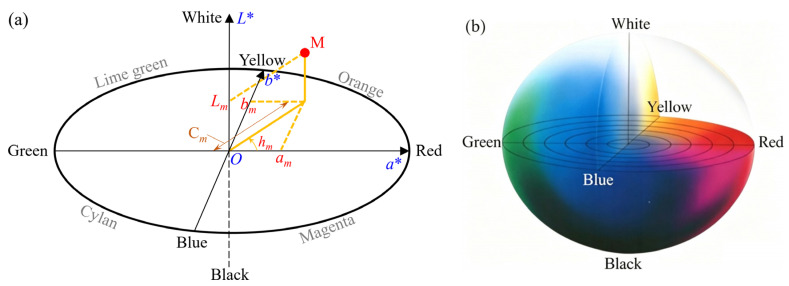
Schematic diagram of the physical meaning of color quantification parameters in a color space coordinate system. (**a**) Diagram of color parameters [45,46,47]; (**b**) color distribution within the color space [48].

The color parameters measured by the colorimeter include lightness L*, chromaticity components a* and b*, chroma C*, and hue angle *h*. The physical meanings of these optical parameters are illustrated in Figure 3 [45,46,47,48]. As shown in Figure 3a, in the color space coordinate system, *O* represents the origin. The horizontal axis, vertical axis, and perpendicular axis correspond to the red–green chromaticity component (a*), yellow–blue chromaticity component (b*), and lightness (L*), respectively. The value of chroma C* is equal to $\sqrt{a^{*2}+b^{*2}}$ [42,46]. As a simple example, for point M in Figure 3a, the corresponding lightness, red–green chromaticity component, yellow–blue chromaticity component, chroma, and hue angle are *L_m_*, *a_m_*, *b_m_*, C*_m_*, *h_m_*, respectively. These parameters can be used to quantitatively represent the position of any color within the color space coordinate system, with each specific color corresponding to a unique coordinate set.

**Figure 4:**
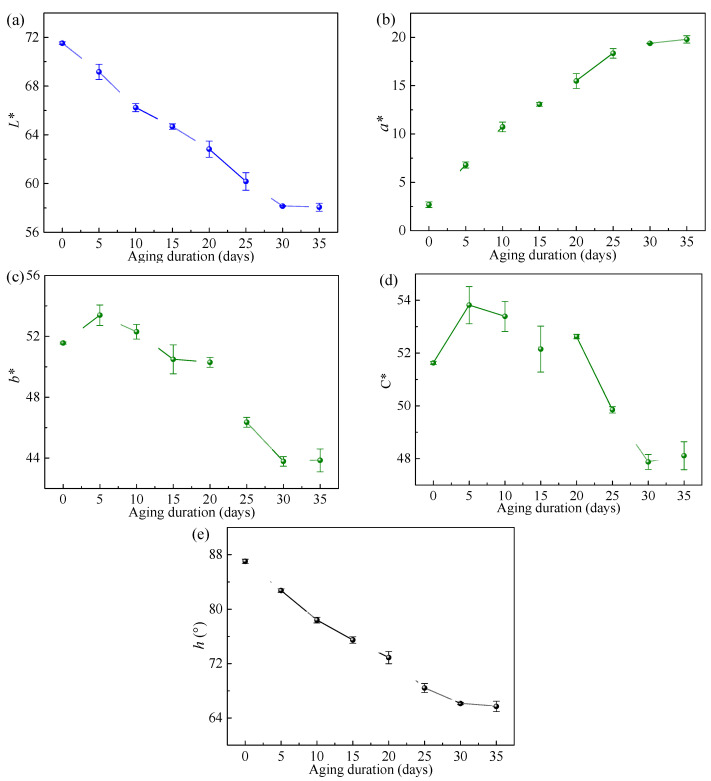
Evolution of color parameters with thermo-oxidative aging duration. (**a**) L* versus aging duration; (**b**) a* versus aging duration; (**c**) b* versus aging duration; (**d**) C* versus aging duration; (**e**) *h* versus aging duration.

The specific measured color parameters of the epoxy resin are listed in Table 1, and the evolution of these parameters with thermo-oxidative aging duration is presented in Figure 4. As observed in this figure, with increasing aging duration, the lightness (L*), the red–green chromaticity component (a*), and the hue angle (*h*) exhibited distinct monotonic trends. Although local fluctuations were present in the yellow–blue chromaticity component (b*) and chroma (C*), their overall trend showed a decrease with prolonged aging. Since the magnitude of a* is considerably smaller than that of b*, and given that $C*=\sqrt{a^{*2}+b^{*2}}$ [42,46], the value of b* predominantly governs the value of C*. Specifically, the decrease in L* with aging indicates a color shift from white to black. The increase in a* and the decrease in *h* suggest a color transition from yellow to red. Meanwhile, the reduction in b* reflects a shift from yellow to blue. These changes collectively indicate that thermo-oxidative aging promotes a transition in the epoxy resin’s color from bright to dark, which aligns with the visually observed color changes presented in Figure 2.

### 3.2. Evolution of Mechanical Properties with Thermal-Oxidative Aging Duration

**Figure 5:**
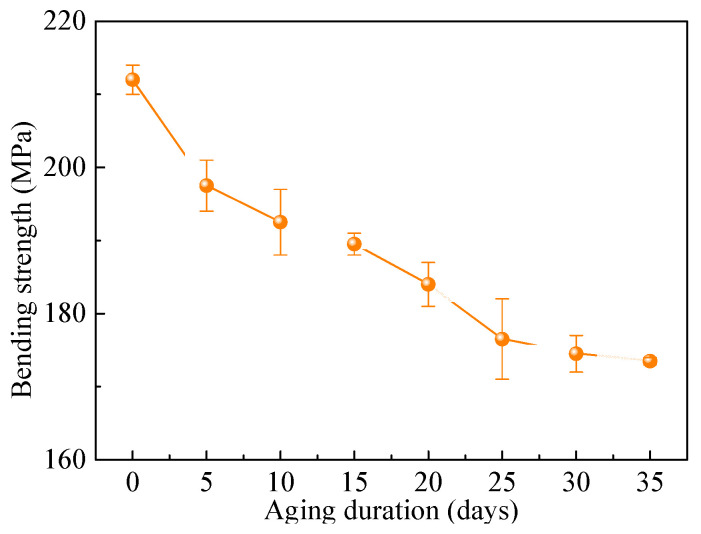
Evolution of three-point bending strength with thermal-oxidative aging duration.

Figure 5 shows the variation in the three-point bending strength of the epoxy resin specimens with thermo-oxidative aging duration. From this figure, the strength of the epoxy resin gradually decreases with increasing aging duration. This observed trend is consistent with findings from other studies on the thermo-oxidative aging behavior of epoxy resins [37,38].

### 3.3. Quantitative Relationship Between Color Parameters and Mechanical Properties

**Figure 6:**
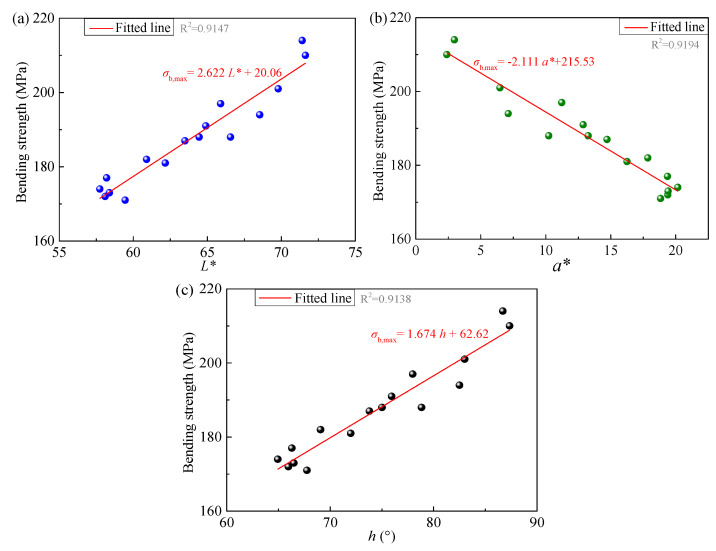
Relationship between color parameters and bending strength of epoxy resin during thermo-oxidative aging in this work. (**a**) L* versus bending strength; (**b**) a* versus bending strength; (**c**) *h* versus bending strength.

On the basis of clarifying the evolution trends of both color parameters and bending strength of the epoxy resin with thermo-oxidative aging duration, a relationship between the color parameters and the bending strength can be established. As the objective of this study is to provide a method for estimating mechanical properties using polymer color parameters, this process requires ensuring that a specific color value corresponds to a unique bending strength value. Since the color parameters b* and C* presented in Figure 4 exhibit non-monotonic evolution with aging duration, while the bending strength in Figure 5 shows a monotonic decreasing trend, it is theoretically infeasible to estimate the bending strength using b* and C*. Conversely, the color parameters L*, a*, and *h* shown in Figure 4 all change monotonically with increasing aging time. Therefore, in theory, a one-to-one mapping relationship can be established between each of these three color parameters and the bending strength. As illustrated in Figure 6, the relationships between L*, a*, *h* and the bending strength (*σ_b_*_,max_) exhibit approximately linear patterns. Consequently, they can be fitted using the following linear equations

(1) $$\sigma_{b,\max}=p_{1}L*+p_{2}$$

(2) $$\sigma_{b,\max}=p_{3}a*+p_{4}$$

(3) $$\sigma_{b,\max}=p_{5}h+p_{6}$$

where *p*_1_, *p*_2_, *p*_3_, *p*_4_, *p*_5_, *p*_6_ are all constants determined through fitting. For the epoxy resin used in this study, the constant values calculated by least-squares fitting, as shown in Figure 6, are *p*_1_ = 2.622, *p*_2_ = 20.06, *p*_3_ = −2.111, *p*_4_ = 215.53, *p*_5_ = 1.674, *p*_6_ = 62.62. As shown in Figure 6, the R^2^ statistics (coefficient of determination [49]) for the fittings using L*, a*, and *h* are 0.9147, 0.9194, and 0.9138, respectively. These three values are very close to each other. This indicates that using any of these three color parameters to fit the strength of the resin used in this experiment yields fitting results with comparable accuracy. It should be noted that if the relationship between color parameters and bending strength for certain polymer is not linear, other forms of curve-fitting equations, such as power functions or piecewise functions, may be considered. Whether this relationship holds for other aging conditions or different polymers requires rigorous scientific verification.

**Figure 7:**
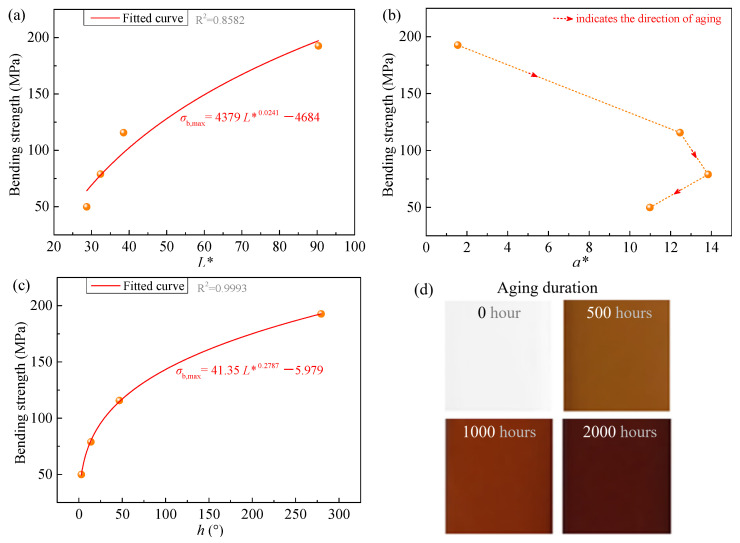
Relationship between color parameters and strength of epoxy resin samples during aging as reported in Ref. [37], under conditions of 120 °C in air. (**a**) L* versus bending strength; (**b**) a* versus bending strength; (**c**) *h* versus bending strength; (**d**) sample photographs. Note: Sample photographs and strength data are from Ref. [37], color parameters were measured by us using a colorimeter according to the color of sample photographs. The reuse of (d) has been licensed by Elsevier (see Appendix B for details).

**Figure 8:**
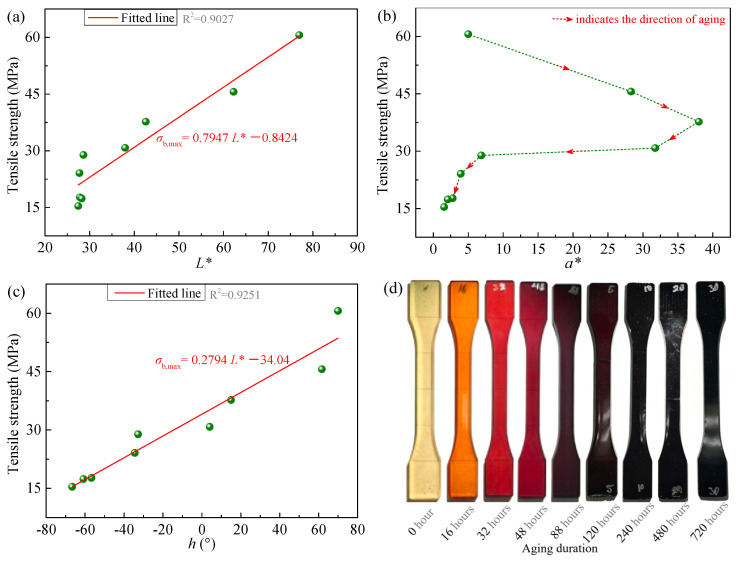
Relationship between color parameters and strength of epoxy resin samples during aging as reported in Ref. [38], under conditions of 160 °C in air. (**a**) L* versus tensile strength; (**b**) a* versus tensile strength; (**c**) *h* versus tensile strength; (**d**) sample photographs. Note: Sample photographs and strength data are from Ref. [38], color parameters were measured by us using a colorimeter according to the color of sample photographs. (d) reused has been licensed by Elsevier (see Appendix B for details).

Furthermore, to examine whether the relationship between color parameters and strength observed in this work has broader applicability, color parameters of epoxy resin samples subjected to different thermo-oxidative aging durations in the Refs. [37,38] were measured, as listed in Table 2, and quantitative relationships between the measured color parameters and strength were established. The results are shown in Figure 7 and Figure 8. From these figures, it can be observed that both L* and *h* of the epoxy resins reported in the literature exhibit monotonic variations with strength. Specifically, strength increases with higher L* or *h* values. However, while L* and *h* in Figure 8a,c show an approximately linear relationship with strength (hence fitted using Equations (1) and (3)), Figure 7a,c reveal a nonlinear, approximately power-law trend (thus fitted using a power function with form of Equation (4)). Regarding a*, as shown in Figure 7b and Figure 8b, it first increases and then decreases with aging time, corresponding to an initial negative and then positive correlation with strength. This differs from the monotonic evolution of a* with aging time and strength observed in Figure 4b and Figure 6b. This discrepancy may be attributed to differences in the extent and stage of aging. The aging conditions in our experiments did not reach the same level as those in Refs. [37,38]. Therefore, a* in Figure 4b and Figure 6b only exhibited monotonic variation without showing an inflection point. Overall, combining our experimental results with those from the literature suggests that the monotonic relationship between L* or *h* and strength appears to be generally observed.

(4) $$\sigma_{b,\max}=q_{1}a{*}^{q_{3}}+q_{2}$$

where *q*_1_, *q*_2_, *q*_3_ are all constants calculated through fitting. If the exponent *q*_3_ = 1, this equation reduces to linear form express as Equation (2).

In addition, we clarify the fitting accuracy for Figure 7 and Figure 8. Regarding Figure 7, the fitting result using *h* is clearly superior to that using L*. However, because the sample size for this group is extremely limited—only four data points, the possibility that the accuracy is constrained by the small sample size cannot be ruled out. As for Figure 8, both L* and *h* exhibit good fitting accuracy, with only a small difference between them.

Here, we briefly describe the procedure for measuring the color parameters of samples from Refs. [37,38] using a colorimeter. Since the actual samples from the literature were not available, color photos of these samples provided in the literature were printed onto standard copy paper using a color printer. The color parameters of the printed photos were then measured with the colorimeter. Although this approach may introduce minor deviations, it does not affect the overall trends and patterns.

### 3.4. Feasibility of Estimating Mechanical Properties Using Color Parameters

**Figure 9:**
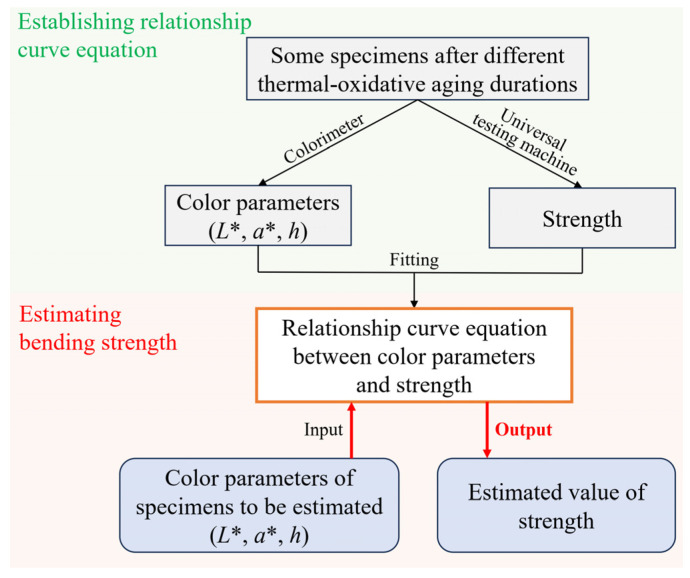
Procedure for estimating strength using colorimetric parameters.

Figure 9 illustrates the procedure for estimating the strength of the epoxy resin specimens used in this work based on color parameters. The specific steps are as follows:

(1) Measure the color parameters of representative specimens subjected to different aging durations using a colorimeter, including L*, a*, and *h*. These three parameters each exhibit a monotonic relationship with strength, as detailed in Figure 6. Subsequently, determine the strength of these specimens using a mechanical testing machine.
(2) Based on the obtained color parameters and strength data from the representative specimens, fit the estimation Equations (1)–(3) to determine the specific constant values.
(3) For a specimen whose strength is to be estimated, measure its color parameter L*, a*, or *h* with the colorimeter. Substitute the measured value into the fitted Equations (1)–(3) and solve to calculate the estimated strength value.

**Figure 10:**
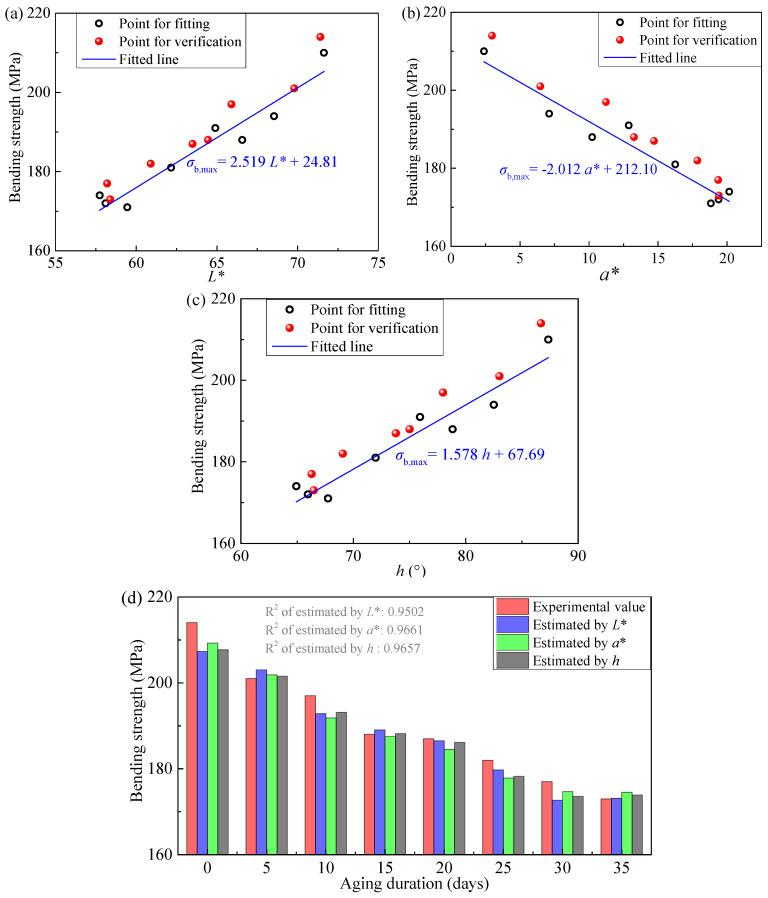
Results of bending strength estimation using colorimetric parameters. (**a**–**c**) show the fitting curves and scatter plots for estimating bending strength using L*, a*, and *h*, respectively; (**d**) presents the specific estimated values. Note: Data from specimens listed in Table 1, specimens #01, #11, #21, #31, #41, #51, #61, #71 were used for fitting, while specimens #02, #12, #22, #32, #42, #52, #62, #72 were used for validation.

Our envisioned practical application of this estimation method is as follows: first, establish the relationship between color parameters and aging duration using low-cost, small-sized samples with different aging durations, which serves as a “calibration curve” for subsequent estimation. Then, use a colorimeter to measure the color parameters of an actual epoxy resin component, and substitute the measured values into the calibration curve to estimate the aging degree and strength of the component. Given the limited number of specimens available in this work and in Ref. [38], the specimens used for fitting and estimation were selected based on the principle of balancing total specimen size with fitting accuracy. For the specimens listed in Table 1, each aging duration includes two specimens (i.e., two sets). Therefore, one set was used to establish the calibration curve (including specimens #01, #11, #21, #31, #41, #51, #61, #71) by fitting Equations (1)–(3), and the other set was used for validation (including specimens #02, #12, #22, #32, #42, #52, #62, #72). The results are presented in Figure 10. From this figure, using the color parameters L*, a*, and *h* to estimate the bending strength demonstrates promising accuracy in each case, with R^2^ statistics of 0.9502, 0.9661, and 0.9657, respectively.

**Figure 11:**
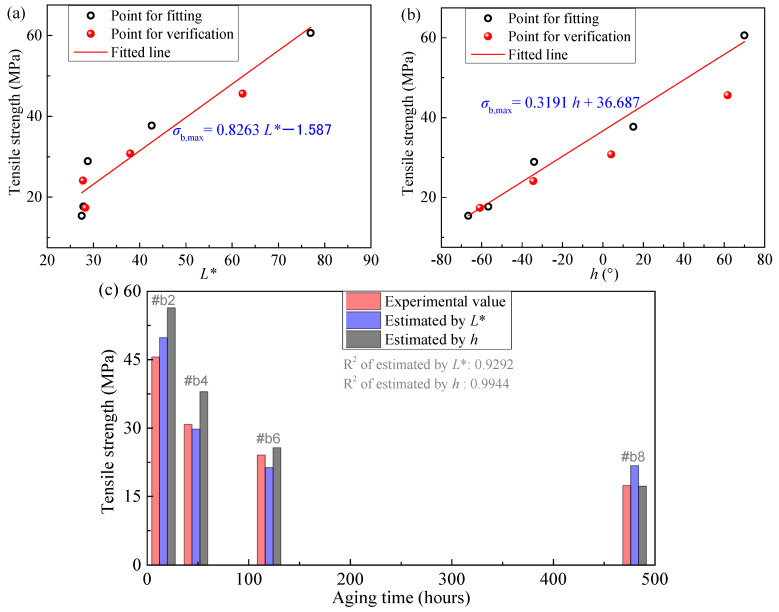
Results of tensile strength estimation using colorimetric parameters. (**a**,**b**) show the fitting curves and scatter plots for estimating tensile strength using L* and *h*, respectively; (**c**) presents the specific estimated values. Note: Data from samples presented in Table 2 and Figure 8, samples #b1, #b3, #b5, #b7, #b9 were used for fitting, while samples #b2, #b4, #b6, #b8 were used for validation.

Additionally, to further demonstrate the general applicability of the proposed estimation method, the data from samples #b1–#b9 in Table 2 and Figure 8 [38] were also used for fitting and validation. Specifically, the parameters of samples #b1, #b3, #b5, #b7, and #b9 were used to fit Equations (1) and (3). Subsequently, samples #b2, #b4, #b6, and #b8 were used to validate the estimation accuracy. The results are shown in Figure 11. A comparison between the experimental and estimated tensile strength presented in this figure indicates that the results still demonstrate satisfactory accuracy. Moreover, the estimation accuracy using *h* is slightly higher than that using L*, which follows the same trend as the fitting accuracy shown in Figure 8. It should be noted that since the parameter a* for this set of samples does not exhibit a monotonic relationship with tensile strength, it cannot be used for estimation, only parameters L* and *h* are applicable in this case.

The validation results above indicate that the empirical method developed in this work, which utilizes color parameters to estimate mechanical properties, is usable and has potential, offering the benefits of low cost and being non-destructive.

## 4. Conclusions

This study experimentally investigated the evolution of color parameters in epoxy resin during thermo-oxidative aging and explored their intrinsic relationship with material strength, leading to the following conclusions.

The thermo-oxidative aging experiments conducted in this study indicate that during the aging process, the strength of the epoxy resin decreases with prolonged aging time, while its color gradually darkens. Specifically, in terms of color space parameters, lightness (L*) and hue angle (*h*) exhibit monotonic decreasing trends. Although local fluctuations are observed in the yellow–blue chromaticity component (b*) and chroma (C*), their overall tendency is to decrease with increasing thermo-oxidative aging duration. As for the red–green chromaticity component (a*), the data from this work show a monotonic increase with aging time, while samples from Refs. [37,38] display a non-monotonic relationship characterized by an initial increase followed by a decrease. This difference may be attributed to the extent and stage of aging.

During thermo-oxidative aging of epoxy resin, clear monotonic relationships were observed between the strength and both lightness (L*) and hue angle (*h*). Furthermore, by utilizing the mechanical performance data of epoxy resin from Refs. [37,38] and indirectly measuring the color parameters of the samples presented therein, the same patterns were identified. The consistency between the experimental findings of the present work and the analysis of independent literature data supports the notion that the correlation between mechanical properties and color in epoxy resin is commonly observed.

Based on the experimental data obtained in this work and the extension of the literature information, an empirical model was proposed for estimating strength using single color parameter—either L*, a*, or *h*. The specific expressions are given in Equations (1)–(3). The comparison between estimated and experimental strengths indicated that the method demonstrates feasibility and high estimation accuracy, while offering the advantages of low cost and being non-destructive.

Although the proposed method can estimate the strength of epoxy resins using their color parameters, it remains empirical. It cannot guarantee that the relationship between each color parameter and strength is monotonic. Moreover, color parameters that do not exhibit a monotonic relationship with strength cannot be used for strength estimation. Therefore, the selection of which color parameter to use for strength estimation should depend on the specific resin characteristics. This is also a limitation of the present method.

## Figures and Tables

**Table 1:** Color parameters of epoxy resin specimens measured by a colorimeter after various thermo-oxidative aging durations.

Aging Duration (Days)	Specimen ID	L*	a*	b*	C*	*h*	Color
0	#01	71.63	2.39	51.63	51.68	87.34	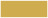
0	#02	71.41	2.97	51.49	51.57	86.69	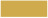
5	#11	68.54	7.11	54.06	54.52	82.50	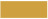
5	#12	69.79	6.47	52.72	53.11	83.00	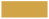
10	#21	66.57	10.23	51.82	52.82	78.83	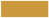
10	#22	65.90	11.23	52.78	53.96	77.98	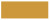
15	#31	64.90	12.88	51.44	53.02	75.94	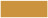
15	#32	64.45	13.26	49.54	51.28	75.01	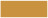
20	#41	62.16	16.25	49.97	52.54	71.98	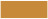
20	#42	63.49	14.71	50.62	52.71	73.79	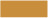
25	#51	59.45	18.83	46.03	49.73	67.75	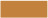
25	#52	60.90	17.85	46.68	49.97	69.07	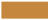
30	#61	58.10	19.39	43.47	47.59	65.96	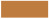
30	#62	58.20	19.36	44.10	48.16	66.29	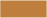
35	#71	57.74	20.16	43.10	47.58	64.93	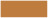
35	#72	58.39	19.41	44.60	48.64	66.48	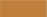

**Table 2:** Color parameters of epoxy resin specimens measured by a colorimeter after various thermo-oxidative aging durations, which specimens cited from Refs. [37,38].

Sample ID	Aging Duration (Hours)	Temperature (°C)	L*	a*	b*	C*	*h*	Color
#a1	0	120	90.36	1.55	−9.30	9.42	279.46	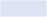
#a2	500	120	38.52	12.46	13.25	18.18	46.75	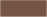
#a3	1000	120	32.38	13.84	3.43	14.25	13.91	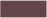
#a4	2000	120	28.70	10.98	0.55	10.99	2.86	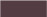
#b1	0	160	76.96	5.00	13.72	14.60	69.97	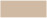
#b2	16	160	62.24	28.30	52.51	59.65	61.67	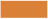
#b3	32	160	42.59	38.00	10.20	39.34	15.02	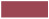
#b4	48	160	37.95	31.76	2.26	31.84	4.07	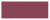
#b5	88	160	28.77	7.05	−4.77	8.51	−34.09	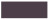
#b6	120	160	27.74	3.93	−2.70	4.76	−34.49	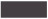
#b7	240	160	27.83	2.78	−4.24	5.07	−56.75	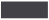
#b8	480	160	28.24	2.09	−3.75	4.29	−60.87	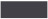
#b9	720	160	27.46	1.55	−3.59	3.91	−66.65	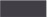

Note: Samples #a1~#a4 are cited from Ref. [37], and samples #b1~#b9 are cited from Ref. [38]. The color parameters in this table were obtained by measuring the colors of sample photographs from the literature.

**Figure A1:**
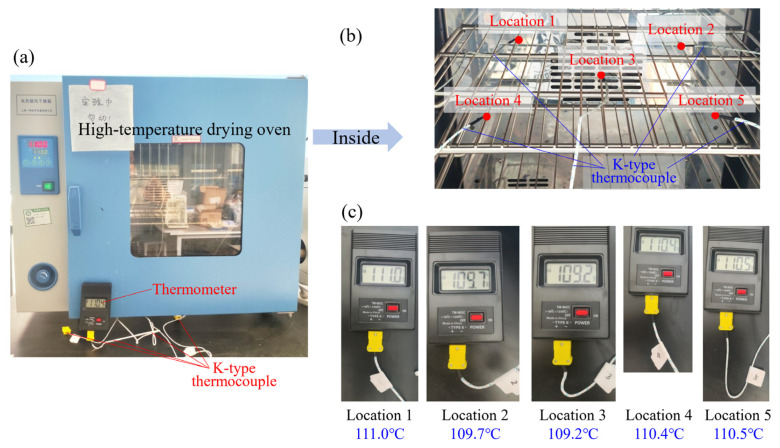
Measurement of temperature error in specimen storage area. (**a**) Overall diagram of the device; (**b**) distribution of temperature measurement locations; (**c**) temperature measurement results corresponding to the five locations.

## Data Availability

The original contributions presented in this study are included in the article. Further inquiries can be directed to the corresponding author.

## Appendix A

To ensure that all specimens experience the same high-temperature environment during aging, the temperature stability of the specimen placement area inside the high-temperature oven was tested. The measured area was approximately 20 cm × 20 cm, located at the same horizontal height as the actual specimen placement. As shown in Figure A1, five K-type thermocouples were installed in this approximately square area and were labeled as Locations 1~5. During the test, the oven door was kept closed. After the temperature reached the set value of 110 °C, a thermometer was connected sequentially to the five thermocouples to measure the temperatures at each location, which measured values were 110.0 °C, 109.7 °C, 109.2 °C, 110.4 °C, and 110.5 °C, respectively. Clearly, the temperature variation across the measured area was within ±1 °C. In fact, the actual specimen placement area is smaller than this measured area; thus, the temperature variation among the specimens is expected to be even smaller. Therefore, we consider it feasible to conduct epoxy resin aging experiments using this high-temperature oven. It should be noted that all specimens were aged at the same height to avoid errors caused by hot air rising to higher positions and cold air settling at lower positions. Additionally, the oven is equipped with an internal fan that promotes air circulation, further contributing to a uniform temperature distribution inside the oven.

## Appendix B

In this paper, Figure 7d and Figure 8d are cited from Refs. [37] and [38], respectively. In accordance with the principle of respecting intellectual property rights, these figures reuse has been licensed by Elsevier, the publisher of both references. License details are provided in Table A1.

In addition, in Ref. [38], the aging duration of some samples is given in unit of hours, while that of others is given in unit of days. After citing these data in this paper, the units are uniformly converted to hours, as present in Figure 9 and Table 2.

**Table A1:** Reuse license information for Figure 7d and Figure 8d of this paper.

Item	Specific Information	
	Figure 7d	Figure 8d
Cited from	Figure 1 in Ref. [37]	Figure 3 in Ref. [38]
License date	30 April 2026	1 May 2026
License number	6259060378850	6260111276293
Licensor publisher	Elsevier	Elsevier
Licensor publication	*Polymer Testing*	*Polymer Degradation and Stability*
Licensor paper title	Effect of thermo-oxidation on the failure properties of an epoxy resin	Experimental characterization and constitutive modeling of bulk epoxy under thermo-oxidative aging
Type of use	Journal/Magazine	Journal/Magazine
Request type	Publisher, not-for-profit	Publisher, not-for-profit
Targeted journal	*Polymers*	*Polymers*
Targeted Publisher	MDPI	MDPI
Targeted article title	Quantitative Relationship Between Color Parameters and Mechanical Property of Epoxy Resin during Thermo-oxidative Aging	
